# Duodenal Atresia

**Published:** 2014-01-01

**Authors:** Vivek Gharpure

**Affiliations:** Department of Pediatric Surgery, Children's Surgical Hospital 13, Pushpanagari, Aurangabad, 431001, India

 (This section is meant for residents to check their understanding regarding a particular topic)

## Questions

1. Briefly describe embryogenesis of duodenal atresia.
2. How duodenal atresia is classified?
3. What anomalies are associated with duodenal atresia?
4. Discuss the role of antenatal ultrasound in babies with duodenal atresia
5. What are the important clinical features of duodenal atresia?
6. What investigations are performed in a neonate suspected to have duodenal atresia?
7. Discuss preoperative stabilization and management of duodenal atresia.
8. How is a windsock deformity treated?
9. What additional postoperative care is necessary in patients with duodenal atresia?
10. How should parents of a baby with duodenal atresia be counseled?

## Answers

1. Intestine goes through a stage of obliteration in the sixth week of intrauterine life. Over next few weeks, intestine recanalizes. Errors of recanalization are considered to be the primary causes of duodenal atresia. Total failure will lead to complete atresia; partial failure may result in stenosis. This is different from atresia of small bowel which is considered to be secondary to a vascular accident.
2. Gray and Skandalakis classified duodenal atresia in three types
  - Type I - these have a web formed by mucosa and submucosa with no defect in the muscle coat. A windsock deformity may occur if the web is thin. Base of the membrane is in second part of duodenum. These constitute 92% case.
  - Type II – duodenal ends are atretic, separated by some distance but attached by a cord. Mesentery is intact. 1%
  - Type III - duodenal ends are atretic, separated by some distance but without any tissue intervening. Mesentery has a V shaped defect. 7%
3. Malrotation, Down’s syndrome, congenital heart disease, anorectal malformations, urinary tract anomalies, esophageal atresia. Many babies with duodenal atresia are low birth weight. Few are associated with multiple anomalies.
4. These days, many babies are diagnosed antenatally. Mother has polyhydramnios in 30-60% cases. Additionally, a large gastric bubble may be seen. Dilated duodenum may also be detected. Distal small bowel may appear smaller than normal. Sonography facilitates early diagnosis, and parents can be counseled about the need for surgical intervention, transfer to another centre. However, outcomes have not changed due to early diagnosis.
5. Early (24-48 hours) onset of bilious vomiting in absence of abdominal distension in a low birth weight child should make one suspect duodenal atresia. These babies may have epigastric fullness.
6. Diagnosis is established by a plain x-ray abdomen. X-ray should be obtained after passing a feeding tube, evacuating the stomach and instilling 40-50 ml air in the stomach to delineate the ‘double bubble’. Lack of gas in distal bowel will indicate complete obstruction. Otherwise partial obstruction should be suspected. Partial obstructions can be difficult to diagnose and may manifest at a later age. In these patients upper GI contrast studies may be necessary. Other investigations like abdominal USG, echocardiography may be performed to detect other congenital anomalies. It is not necessary to perform karyotype in emergency.
7. Duodenal atresia is a comparative emergency but time should be spent in evaluation, stabilization, and rehydration. Stomach and duodenum is decompressed by a small nasogastric tube. Electrolyte losses are corrected. Parenteral nutrition may be started. These patients often have prolonged duodenal ileus.
8. Windsock deformity is treated by opening the duodenum, resecting anterior part of diaphragm with cautry, preserving the ampulla and closing the duodenotomy in transverse fashion. Before closing the duodenum, a large bore catheter should be passed proximally in stomach to ensure complete excision of the membrane.
9. Babies are kept in NICU, given adequate iv fluids/parenteral nutrition. Nasogastric decompression is continued till the drainage is minimum and color changes to pale green. Keeping the patient head up or right decubitus helps duodenal drainage. Once peristalsis is established, small feeds are commenced which are rapidly increased to full strength milk in few days.
10. Babies with duodenal atresia may have other serious congenital anomalies which may alter outcome. Trisomy 21 should be particularly stressed as this can necessitate long term care and rehabilitation. Even after surgical repair, babies may need to stay in NICU for several days/weeks and may require parenteral nutrition with associated complications. Some patients develop obstruction at anastomotic site after many years and may require additional surgery.

## Footnotes

**Source of Support:** Nil

**Conflict of Interest:** None

